# Skeletal muscle autophagy remains responsive to hyperinsulinemia and hyperglycemia at higher plasma insulin concentrations in insulin‐resistant mice

**DOI:** 10.14814/phy2.13810

**Published:** 2018-07-25

**Authors:** Sarah E. Ehrlicher, Harrison D. Stierwalt, Sean A. Newsom, Matthew M. Robinson

**Affiliations:** ^1^ College of Public Health and Human Sciences Oregon State University Corvallis Oregon

**Keywords:** Macroautophagy, mechanistic target of rapamycin, mitochondria, obesity, protein degradation

## Abstract

Skeletal muscle autophagy is suppressed by insulin, but it is not clear if such suppression is altered with insulin resistance. We investigated if the inhibitory action of insulin on autophagy remains intact despite insulin resistance to glucose metabolism. C57BL/6J mice consumed either a low‐fat (10% fat) diet as control or high‐fat (60% fat) diet for 12 weeks to induce insulin resistance. Following a 5‐hour fast, mice underwent either hyperinsulinemic‐euglycemic, hyperinsulinemic‐hyperglycemic, or saline infusion to test the effect of insulin on autophagy markers in the quadriceps muscle (*n *= 8–10 per diet and clamp condition). Mice were anesthetized by sodium pentobarbital for tissue collection after 2 h of infusion. Despite the high‐fat group having lower insulin‐stimulated glucose uptake, both low‐fat and high‐fat groups had similar autophagosome abundance during hyperinsulinemic conditions. The lipidation of microtubule‐associated proteins 1A/1B light chain 3B (LC3II/LC3I) was decreased in hyperinsulinemia versus saline control (*P* < 0.01) in low‐fat (−54%) and high‐fat groups (−47%), demonstrating similar suppression of autophagy between diet groups. Mitochondrial‐associated LC3II was greater in the high‐fat compared to the low‐fat group (*P* = 0.045) across clamp conditions, suggesting a greater localization of autophagosomes with mitochondria. L6 myotubes were treated with insulin and rapamycin to determine the role of mechanistic target of rapamycin complex‐1 (mTORC1) in insulin‐mediated suppression of autophagy. Inhibition of mTORC1 blunted the decline of LC3II/LC3I with insulin by 40%, suggesting mTORC1 partially mediates the insulin action to suppress autophagy. Collectively, autophagy remained responsive to the suppressive effects of insulin in otherwise insulin‐resistant and obese mice.

## Introduction

Autophagy is a catabolic pathway by which cellular components are degraded in lysosomes. Autophagy is distinguished into more targeted micro and chaperone‐mediated autophagy (Cuervo and Dice 1996), or bulk degradation through macroautophagy whereby cellular components are engulfed by autophagosomes that fuse to lysosomes for degradation (Singh and Cuervo 2011). The induction of autophagosome formation is a primary mechanism for the removal of damaged or dysfunctional proteins, particularly of the mitochondria, to maintain protein homeostasis in skeletal muscle (Masiero et al. 2009). Disruption of signaling to induce autophagy can impede remodeling of the skeletal muscle proteome and mitochondrial network in response to stress or injury (Call et al. 2017).

Autophagy is sensitive to nutritional signals and can be suppressed by increased concentrations of insulin (Naito et al. 2013). The development of insulin resistance with obesity is characterized by decreased insulin action in skeletal muscle and decreased glucose uptake in response to insulin (Rizza et al. 1981b). Hyperinsulinemia with obesity helps compensate for attenuated glucoregulatory actions of insulin (Firth et al. 1987), but may also impact the regulation of autophagy. For example, adults with type 2 diabetes and hyperinsulinemia appear to have suppressed autophagosome formation and clearance in skeletal muscle compared to lean adults (Møller et al. 2017). Another study indicated that insulin infusion during euglycemia below fasting failed to decrease autophagy signaling in adults with type 2 diabetes until glucose was elevated to fasting concentrations (Kruse et al. 2015). It is possible that autophagy signaling is decreased with insulin resistance, but it is not clear if suppression is due to an intrinsic defect in autophagy or a response to hyperinsulinemia.

Autophagy is a contributor to skeletal muscle protein degradation and examples of a maintained protein degradation response to insulin have been detected with insulin resistance. For example, obese adults with fasting hyperinsulinemia had lower rates of protein degradation when measured during basal conditions compared to lean controls (Denne et al. 1995). Hyperinsulinemic clamp conditions resulted in suppression of protein degradation when measured at the whole body (Pereira et al. 2007) and across the leg (Bell et al. 2006) in insulin‐resistant and insulin‐sensitive adults. Such findings indicate that protein degradation remains responsive to insulin. Declines in autophagy‐mediated protein degradation may not be derived from defective autophagy machinery, but instead from a response to chronic hyperinsulinemia.

A potential mechanism for suppression of autophagy with insulin resistance is through activation of the mechanistic target of rapamycin complex 1 (mTORC1), a primary inhibitor of autophagy that is activated by insulin (Kim et al. 2011). Obesity results in greater activation of mTORC1 (Khamzina et al. 2005) and infusion of insulin stimulates activation of mTORC1 by phosphorylation at Ser^2448^ (Timmerman et al. 2010). It is therefore possible that declines in autophagy activation with insulin resistance are a response to hyperinsulinemia and greater mTORC1 activity.

The purpose of this study was to determine if the inhibitory action of insulin on autophagy remains intact during insulin resistance. The rationale was that suppression of autophagy by insulin may contribute to decreased protein degradation with obesity as a result of chronic hyperinsulinemia. A downstream consequence of decreased protein degradation could be the accumulation of damaged and dysfunctional proteins, particularly of mitochondria. We hypothesized that skeletal muscle autophagy would be suppressed during hyperinsulinemic conditions to a similar extent in lean mice and mice with diet‐induced obesity. High‐fat diet‐induced insulin‐resistant mice were studied during hyperinsulinemic/euglycemic or hyperinsulinemic/hyperglycemic conditions compared to saline control to test the suppressive effects of higher insulin concentrations on autophagy. We further hypothesized that with insulin resistance, mTORC1 activation contributes to the suppression of autophagy in response to high concentrations of insulin, as measured in mice and L6 myotubes. The main finding was that insulin‐resistant mice remained responsive to the inhibitory action of insulin on autophagy activation, and cell culture experiments demonstrate the suppression of autophagy was in part due to intact signaling through mTORC1.

## Materials and Methods

### Animal care and study design

This study was approved by the Animal Care and Use Committee at Oregon State University (#4788). Twelve‐week‐old male C57BL/6J mice (Jackson Laboratories) were housed 3–5 per cage under standard 12‐h light–dark cycle at 22°C conditions with free access to food and water. Mice consumed either a low‐fat diet (LFD, *n* = 23) (D12450J; Research Diets, New Brunswick, NJ) or high‐fat diet (HFD, *n* = 28) (D12492; Research Diets, New Brunswick, NJ) for 12 weeks. The percent kilocalories from total fat (from lard)/carbohydrate/protein was 10/70/20 for the LFD and 60/20/20 for the HFD. In‐cage metabolic assessments (Promethion, Sable Systems Int., Las Vegas, NV) were performed at week 10 along with body composition by nuclear magnetic resonance spectroscopy (EchoMRI, Houston, TX). At week 12, mice were fasted for 5 h then underwent one of three different infusion protocols to alter insulin and/or glucose concentrations: a saline infusion with maintained fasting insulin (low) and euglycemia (SAL), a hyperinsulinemic‐euglycemic clamp (HI/EG), or a hyperinsulinemic‐hyperglycemic clamp (HI/HG). The HI/EG and HI/HG clamps were performed to measure whole‐body insulin sensitivity and skeletal muscle autophagy markers under a controlled nutrient stimulus. The SAL clamp served as a nonstimulated condition to determine basal levels of autophagy markers and to account for potential effects following the surgical catheterization performed on each mouse prior to clamp conditions (described below). Immediately after the infusion, mice were anesthetized by sodium pentobarbital and tissues were freeze‐clamped for further analysis.

### Metabolic assessment

Total and resting energy expenditure was measured over a 72‐h period at week 10 by continuous metabolic monitoring (Promethion, Sable Systems Int., Las Vegas, NV). Animals were singly housed in the metabolic chamber system and allowed to acclimate for 24 h before data collection. Whole‐body energy expenditure and substrate oxidation was determined by indirect calorimetry and calculated based on oxygen consumption and carbon dioxide production (Lark et al. 2018). During the metabolic assessment, physical activity was continuously measured by a multidimensional beam break system and food intake and body weight were continuously measured by electronic scales.

### Surgery and clamps

All procedures required for the clamp conditions were approved by the Animal Care and Use Committees at Oregon State University and Vanderbilt University. Catheters were implanted into a carotid artery and a jugular vein of mice for sampling and infusions, respectively, 5 days before the study as described previously (Berglund et al. 2008). The clamps were performed on mice fasted for 5 h as described previously (Ayala et al. 2006) and were modified for the specific infusion. Arterial glucose was clamped using a variable infusion rate of glucose, which was adjusted based on the measurement of blood glucose at 10 min intervals. Blood glucose was clamped at ~110 mg/dL for the HI/EG clamp and at 350 mg/dL for the HI/HG clamp. For the HI/EG clamp, at time zero, insulin infusion (4 mU/kg of body weight per min) was started and continued for 120 min. In all clamp conditions, mice received heparinized saline‐washed erythrocytes from donor mice at 5 *μ*L/min to prevent a fall in hematocrit. Blood samples were collected from the carotid artery at *t* = −10, 100 and 120 min for determination of plasma insulin. Plasma insulin was determined by radioimmunoassay.

### Cell culture

L6 myoblasts (CRL‐1458; ATCC, Manassas, Virginia, USA) were grown in Dulbecco's modified Eagle's medium (DMEM) supplemented with 1% antibiotic/antimycotic (AbAm) and 10% fetal bovine serum (FBS) in a 5% CO_2_‐humidified atmosphere at 37°C and seeded to 6‐well plates. Once cells reached ~80–90% confluence, differentiation into myotubes was induced by switching to low glucose DMEM supplemented with 1% AbAm and 2% horse serum for 5–6 days.

To model insulin resistance, myotubes were treated with 300 *μ*mol/L palmitate (palmitic acid; Sigma‐Aldrich, St. Louis, MO, USA) dissolved in 100% ethanol and prepared in culture media supplemented with 2% bovine serum albumin and 1 mmol/L carnitine for 18 h prior to insulin stimulation. The palmitate media were removed and myotubes were incubated in serum‐free media (low glucose DMEM + 1% AbAm) concurrently with insulin for 4 h or serum‐free media and no insulin for 4 h as a control condition. Insulin doses included a low dose (0.5 nmol/L) and a high dose (100 nmol/L) of insulin (bovine insulin; Sigma‐Aldrich, St. Louis, MO, USA). Autophagosome degradation was inhibited with 100 nmol/L bafilomycin (Sigma‐Aldrich, St. Louis, MO, USA) treatment for the duration of the insulin stimulus. To inhibit mTORC1, myotubes were incubated for 4 h in serum‐free media concurrently with 10 nmol/L rapamycin (no. 9904; Cell Signaling Technology) then insulin (100 nmol/L) was added for the final 2 h of the experiment. Cells were harvested in 250 *μ*L of lysis buffer plus protease inhibitor cocktail, centrifuged (10 min at 1000*g*) and the supernatant was collected for immunoblotting.

### Immunoblotting

Quadriceps muscle was homogenized as whole tissue (~30 mg) and mitochondrial isolations (~80 mg) for immunoblotting as previously described (Newsom et al. 2017). Approximately 35 *μ*g of protein from tissue or cell lysates was resolved on 7–12% Bis‐Tris gels and transferred to nitrocellulose membranes. Two control samples (20 *μ*g of insulin‐stimulated L6 cell samples) were loaded onto each gel and their average intensity was used to normalize intensities between gels. Ponceau staining of membranes was used to verify equal loading and transfer of protein to the nitrocellulose membrane. Images were generated using infrared detection (Odyssey, Licor, Lincoln NE). Primary antibodies included Akt (no. 2920; Cell Signaling Technology), pAkt^Ser473^ (no. 9271; Cell Signaling Technology), LC3 (no. 12741; Cell Signaling Technology), p62 (no. 7695; Cell Signaling Technology), mTOR (no. 4517; Cell Signaling Technology), pmTOR^Ser2448^ (no. 2971; Cell Signaling Technology), pUlk1^Ser757^ (no. 6888; Cell Signaling Technology), AMPK (no. 5832; Cell Signaling Technology), pAMPK^Thr172^(no. 2535; Cell Signaling Technology). Secondary antibodies included anti‐rabbit‐700 (1:10,000; no. 926‐68071; Licor) and anti‐mouse‐800 (1:50,000; no. 926‐32212; Licor).

### Quantitative polymerase chain reaction (qPCR)

Total mRNA was extracted and analyzed by qPCR as previously reported (Robinson et al. 2016). Total mRNA from mouse quadriceps muscle using an RNeasy kit (Qiagen Inc, Valencia, CA, USA) then purity and concentration determined using a NanoDrop spectrophotometer (Thermo Fisher Scientific Inc., DE, USA). One microgram of mRNA was reverse transcribed to cDNA (Life Technologies Corp, CA, USA). Forty‐five nanograms of cDNA was amplified using Taqman^®^ reagents and predesigned gene expression assays FAM^®^ labeled probes (Life Technologies Corp, NY, USA) for p62 (Mm00448091_m1) and LC3 (Mm00458724_m1) with 18s (#4318839, VIC^®^ labeled) as reference gene. Singleplex qPCR (ABI 7900, Thermo Fisher Scientific Inc., DE, USA) was performed with target and reference genes amplified in 20 *μ*L on 384 well clear plates with 40 cycles of 15 sec denaturing (95°C) and 60 sec annealing/extension (60°C). A no template control and internal repeated control was included on every plate. Relative quantification was performed using an 8‐point relative standard curve spanning 2 log dilutions with the target gene normalized to the reference gene. Similar amplification efficacies (97–99%) were verified between target and references genes.

### Statistical analyses

Energy expenditure was analyzed by analysis of covariance (ANCOVA) with lean mass as a correlative variable. Clamp variables and autophagy markers were analyzed with a two‐way analysis of variance model (ANOVA) by diet and clamp conditions. Statistical significance was set as *P* < 0.05. Statistical analysis was performed using Prism version 6 (GraphPad Software, La Jolla, CA). Our previous investigations in similar mice and diet interventions reported LC3‐II abundance by western blotting had a mean of 0.191 and standard deviation of 0.07 from 10 high‐fat fed mice (Newsom et al. 2017). Using these measures of variance and significance at *α* = 0.05, power calculations indicated that group sizes of *n* = 8 could detect differences of 0.11 (at 1 – *β* = 0.8) and 0.07 (at 1 – *β* = 0.5). These differences are effect sizes of 1.57 and 1.0, respectively, which were considered physiologically relevant for the primary outcome of autophagy signaling. Data are presented as mean and standard deviation, unless otherwise specified.

## Results

### Verification of model and phenotypic data

Mice were fed either a low‐fat control diet (LFD) or a high‐fat diet (HFD) to induce obesity and insulin resistance prior to clamp conditions. After 10 weeks of feeding, body composition and metabolic variables were measured in a subset of the mice (*n* = 8 per diet group of the hyperinsulinemic‐euglycemic group) to verify the model and identify potential differences in physical activity which may impact autophagy signaling. The HFD group had greater percent adiposity, lower average respiratory exchange ratio, and reduced activity in both the light and dark cycles compared to the LFD group (Table 1). Regression analysis demonstrated HFD mice had similar energy expenditure compared to LFD (*P* = 0.57) and both diets groups had a similar relationship between energy expenditure and lean mass (*P* = 0.96).

**Table 1:** Characteristics of mice prior to hyperinsulinemic‐euglycemic clamp

	LFD	HFD	*P* value
Total mass (g)	28.3 (1.69)	39.6 (4.4)	<0.001
Lean mass (g)	18.6 (1.1)	20.8 (1.16)	0.002
Fat mass (g)	3.2 (1.1)	12.0 (4.47)	<0.001
Lean mass (%)	66 (4.19)	53.1 (6.31)	<0.001
Adiposity (%)	11.3 (3.64)	29.6 (8.52)	<0.001
RER light cycle	0.83 (0.08)	0.77 (0.02)	0.04
RER dark cycle	0.89 (0.07)	0.78 (0.02)	<0.001
Food intake (kcal) light cycle	0.95 (0.56)	2.34 (1.84)	0.059
Food intake (kcal) dark cycle	4.11 (1.11)	5.74 (2.44)	0.11
Total activity (m) light cycle	42.7 (5.3)	32.7 (6.8)	0.006
Total activity (m) dark cycle	178.7 (36.3)	124.1 (38.9)	0.01

*P* values are from unpaired *t*‐tests for low‐fat diet (LFD) compared to high‐fat diet (HFD). Data are mean (SD) with *n* =  8 per diet group.

LFD, Low‐fat diet; HFD, High‐fat diet; RER, Respiratory exchange ratio; g, grams; kcal, kilocalories; m, meters.

**Figure 1:**
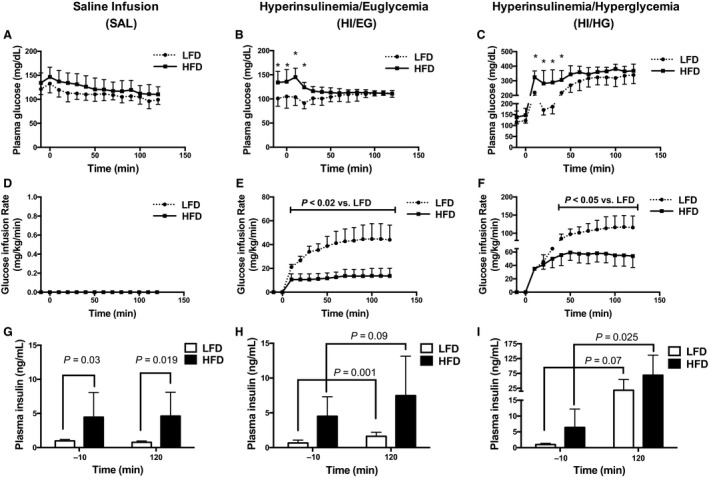
Conditions of the infusion protocols in mice. (A–C) Plasma glucose levels were maintained at similar levels for the low‐fat diet (LFD) and the high‐fat diet (HFD) groups. (D) The SAL infusion did not include a glucose infusion. (E–F) The rate of glucose infusion was greater in the LFD compared to the HFD group. (G) Plasma insulin was elevated throughout the SAL clamp in the HFD group compared to the LFD group. (H–I) Plasma insulin increased in response to the infusion protocols. Panels A–F were analyzed by two‐way ANOVA corrected with Sidak multiple comparison test, *P < 0.05 versus LFD. Panels G–I were analyzed by unpaired *t*‐test. Data are expressed as means and SD,* n* = 7–10 per group.

Whole‐body insulin resistance was confirmed in the HFD group as demonstrated by a threefold reduced glucose infusion rate during HI/EG clamp and a twofold reduced glucose infusion rate during the HI/HG clamp to maintain steady plasma glucose concentrations compared to the LFD group (Fig. 1E and F). The elevated plasma insulin throughout the SAL clamp in the HFD group compared to the LFD group further confirms obesity‐induced insulin resistance (Fig. 1G). Plasma insulin was raised moderately during the HI/EG clamp compared to levels at time ‐10 in the LFD (~2.5‐fold) and HFD (~1.7‐fold) groups and raised even higher in the HI/HG clamp in the LFD (~18‐fold) and the HFD (~10‐fold) groups (Fig. 1H and I). Overall, the HFD group had impaired whole‐body glucose regulation and insulin sensitivity compared to the LFD group and the infusions effectively generated graded doses of insulin during euglycemic and hyperglycemic clamps.

### Autophagy markers responded to insulin similarly in LFD and HFD groups

**Figure 2:**
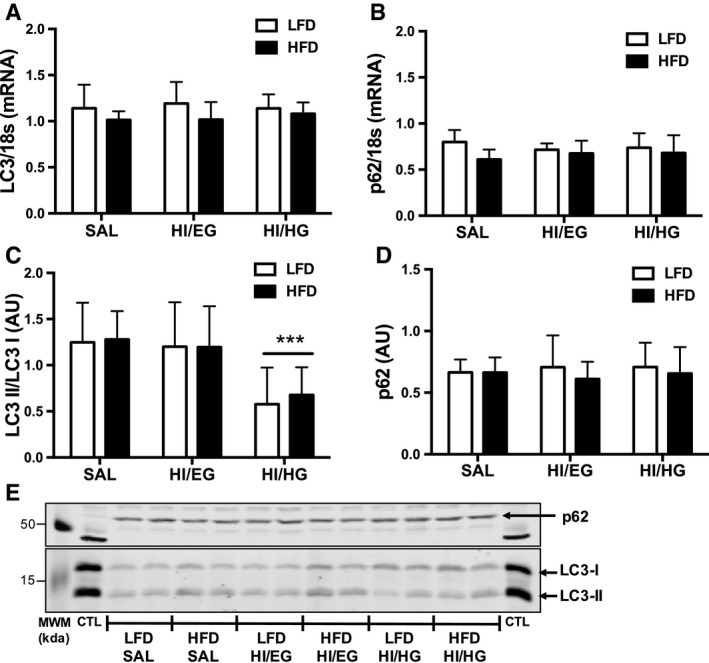
Skeletal muscle mRNA and protein abundance of autophagy markers in mice. (A) Twelve weeks of a high‐fat diet (HFD) lowered LC3 mRNA abundance compared to a low‐fat diet (LFD), with no effect of clamp condition. (B) HFD lowered p62 mRNA abundance compared to LFD, with no effect of clamp condition. (C) LC3II/LC3I protein abundance was suppressed in the HI/HG clamp. (D) p62 protein abundance was not changed by diet or clamp conditions. (E) Representative immunoblots with molecular weight markers (MWM). All samples were derived at the same time and processed in parallel. Total mRNA was extracted from quadriceps and relative quantification performed by quantitative polymerase chain reaction. Data were analyzed by two‐way ANOVA corrected with Tukey's multiple comparison test, *P < 0.05 compared to SAL, **P < 0.05 compared to HI/EG, ***P < 0.05 compared to SAL and HI/EG. Data are expressed as means and SD,* n* = 7–10 per group. AU, arbitrary units.

The quadriceps muscle was analyzed for mRNA and protein abundance of autophagosome markers to measure the autophagy response to insulin. The mRNA abundance of LC3 and p62 were not different at the end of the HI/EG or HI/HG clamps compared to SAL in either diet group (Fig. 2A and B), suggesting that insulin did not alter the transcription of autophagy markers. There was a main effect of diet (*P* < 0.05) for HFD having a lower mRNA abundance of LC3 and p62 compared to the LFD (Fig. 2A and B). The protein abundance of LC3II/LC3I was suppressed in response to the HI/HG clamp in the LFD (−54%) and HFD (−47%) groups compared to the SAL clamp (Fig. 2C), demonstrating reduced autophagosome formation in response to high levels of insulin in both diet groups. The protein abundance of p62 was not different in response to insulin compared to the SAL clamp in either diet group (Fig. 2D).

**Figure 3:**
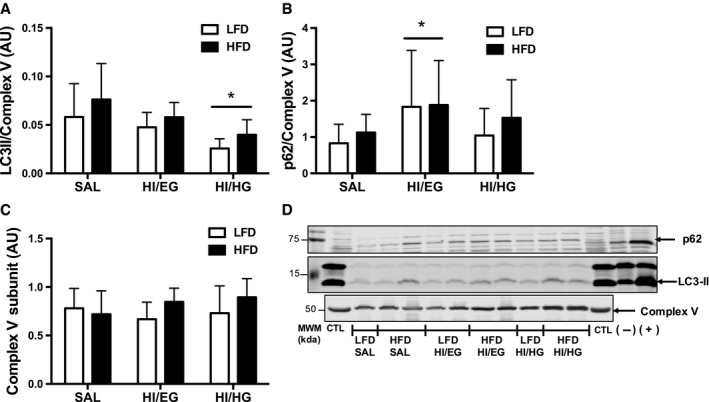
Protein abundance of autophagy markers in a mitochondrial‐enriched fraction of quadriceps muscles in mice. (A) Twelve weeks of a high‐fat diet (HFD) increased LC3II protein abundance compared to a low‐fat diet (LFD). LC3II abundance was suppressed in the HI/HG clamp. (B) p62 abundance was elevated in the HI/EG clamp. (C) Complex V abundance was unchanged by diet or clamp conditions. (D) Representative immunoblots with molecular weight markers (MWM). All samples were derived at the same time and processed in parallel. Data were analyzed by two‐way ANOVA corrected with Tukey's multiple comparison test, *P < 0.05 compared to SAL, **P < 0.05 compared to HI/EG, ***P < 0.05 compared to SAL and HI/EG. Data are expressed as means and SD,* n* = 7–10 per group. AU: arbitrary units.

We next determined potential differences in mitochondrial‐targeted autophagy with insulin resistance. Mitochondrial‐associated LC3II abundance was elevated in the HFD group compared to the LFD group (main effect of diet, *P* = 0.045, Fig. 3A). The abundance of LC3II was reduced in response to the HI/HG clamp in both the LFD (−55%) and HFD groups (−47%) compared to the SAL clamp (Fig. 3A), demonstrating reduced mitochondrial‐associated autophagosome formation in response to high concentrations of insulin. The abundance of p62 was elevated in response to the HI/EG clamp in both the LFD (120%) and HFD groups (67%) compared to the SAL clamp (Fig. 3B), indicating reduced clearance of mitochondrial‐associated autophagosomes in response to moderately elevated insulin. Both diet groups had similar declines in whole tissue and mitochondrial‐associated autophagosome abundance in response to high insulin despite HFD mice having resistance to the glucoregulatory actions of insulin.

### Autophagy signaling remained intact in insulin‐resistant compared to insulin‐sensitive mice

**Figure 4:**
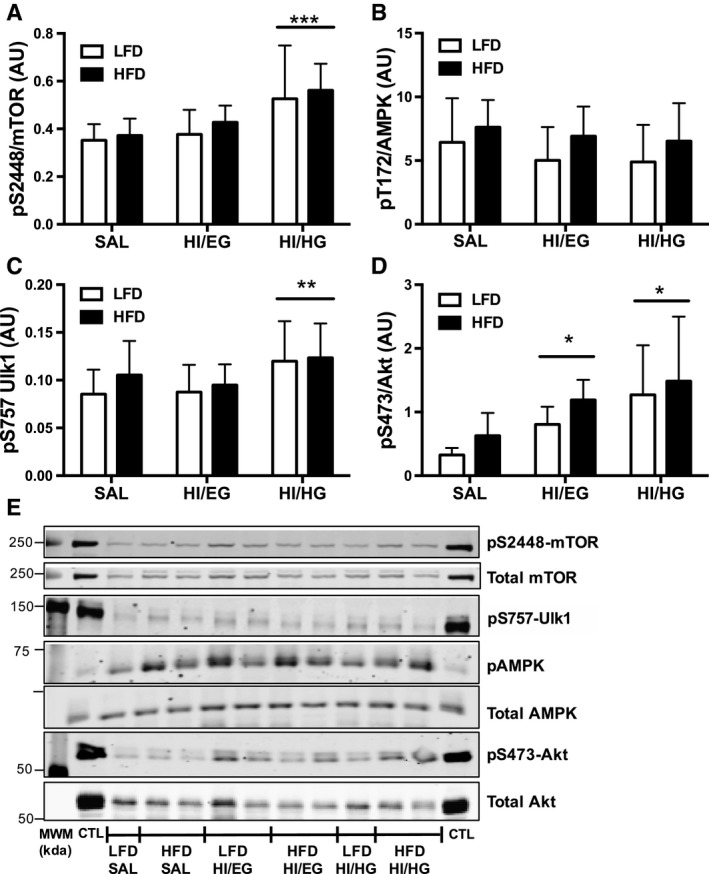
Skeletal muscle signaling in mice. (A) Phosphorylation of mTOR at Ser^2448^ relative to total mTOR was increased in the HI/HG clamp. (B) Phosphorylation of AMPK at Thr^172^ relative to total AMPK was increased with HFD compared to LFD. (C) Phosphorylation of Ulk1 at Ser^757^ was increased in the HI/HG clamp. D) Phosphorylation of Akt at Ser^473^ relative to total Akt was increased in the HI/EG and HI/HG clamps. E) Representative immunoblots with molecular weight markers (MWM). All samples were derived at the same time and processed in parallel. Data were analyzed by two‐way ANOVA corrected with Tukey's multiple comparison test, *P < 0.05 compared to SAL, **P < 0.05 compared to HI/EG, ***P < 0.05 compared to SAL and HI/EG. Data are expressed as means and SD,* n* = 7–10 per group. AU, arbitrary units.

We measured mTORC1 and AMPK as upstream regulators of Ulk1, a primary mediator of autophagosome formation, as mechanisms for autophagy suppression during clamp conditions. In both LFD and HFD groups, mTORC1 phosphorylation at the Ser^2448^ activation site was greater in HI/HG compared to the SAL clamp (Fig. 4A), indicating insulin‐stimulated activation of mTORC1 in both diet groups. Additionally, in response to the HI/HG clamp there was greater phosphorylation of Ulk1 inhibitory site Ser^757^ in the LFD and HFD groups compared to the HI/EG clamp (Fig. 4C), suggesting reduced autophagy activation in response to high levels of insulin. We probed for total Ulk1 and identified weak banding that was not quantifiable (data not shown). There was no effect of clamp conditions on phosphorylation of AMPK at the Thr^172^ activation site (Fig. 4B). The high insulin conditions of HI/EG and HI/HG clamps increased phosphorylation of Akt at Ser^473^ compared to the SAL control in both diet groups (Fig. 4D). Activation of mTORC1 in response to insulin remained intact in the HFD group, which led us to test the requirement of mTORC1 in insulin‐mediated suppression of autophagosome markers.

### Insulin and palmitate impeded autophagosome formation and clearance, respectively, in vitro

**Figure 5:**
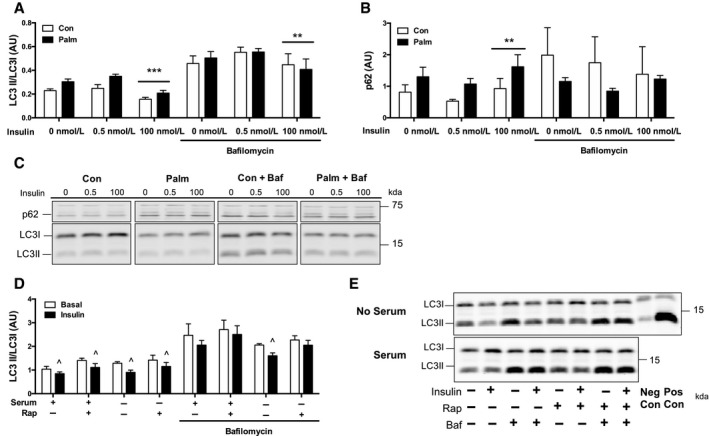
Protein abundance of autophagy markers in L6 myotubes. (A) LC3II/LC3I abundance was suppressed in response to 100 nmol/L insulin treatment in both control (Con) and palmitate (Palm) treated cells (***P < 0.05 compared to 0 nmol/L and 0.5 nmol/L, no Baf). During bafilomycin (Baf) treatment, LC3II/LC3I abundance was suppressed in response to 100 nmol/L insulin treatment in both Con‐ and Palm‐treated cells (**P < 0.05 compared to 0.5 nM insulin with Baf). (B) p62 abundance was elevated in response to 100 nmol/L insulin treatment in both Con‐ and Palm‐treated cells (**P < 0.05 compared to 0.5 nmol/L insulin, no Baf). During Baf, p62 abundance was not different in response to insulin. (C) Representative immunoblots from multiple blots. All samples were derived at the same time and processed in parallel. (D) LC3II/LC3I was suppressed by insulin with and without rapamycin (Rap) treatment. During Baf, LC3II/LC3I was suppressed by insulin without Rap treatment only (^*P* < 0.05 compared to Basal). (E) Representative immunoblots from multiple blots. All samples were derived at the same time and processed in parallel. Panels A and B were analyzed by two‐way ANOVA (Palm × Insulin, with or without Baf) corrected with Tukey's multiple comparison test. Panel D was analyzed by unpaired *t*‐test. Data are expressed as means and SD,* n* = 3 per group. AU, arbitrary units.

Plasma insulin concentrations during clamp conditions were not consistent between the two diet groups, likely because endogenous insulin production was not experimentally restricted. We investigated the effect of controlled insulin doses on autophagy suppression in L6 myotubes made insulin resistant via palmitate exposure. Following 4 h of serum starvation, reintroduction of low‐dose insulin did not alter LC3II/LC3I or p62 abundance in either the control‐ or palmitate‐treated cells (Fig. 5A and B). High‐dose insulin suppressed LC3II/LC3I abundance in both control‐ and palmitate‐treated cells compared to no insulin (Fig. 5A). High‐dose insulin also increased p62 compared to low‐dose insulin (Fig. 5B). Interestingly, there was a main effect of palmitate to increase abundance of LC3II/LC3I (*P* < 0.001) and p62 (*P *< 0.001), suggesting greater autophagosome abundance in the palmitate‐treated cells compared to control cells (Fig. 5A and B). The dose experiments indicated a high insulin concentration was required to alter autophagosome abundance, which was in agreement with the mouse clamp conditions.

We used bafilomycin to inhibit autophagosome degradation to determine autophagosome formation and clearance during the 4‐hour insulin treatment. Bafilomycin treatment resulted in a 66% reduction in the ability of high‐dose insulin to suppress LC3II/LC3I abundance compared to no insulin (Fig. 5A). Bafilomycin prevented the increase in p62 abundance to high‐dose insulin compared to no insulin (Fig. 5B), suggesting that insulin suppresses autophagosome degradation. Interestingly, bafilomycin treatment eliminated the palmitate effect on LC3II/LC3I (main effect of palmitate, *P* = 0.92), whereas p62 was decreased (main effect of palmitate, *P* = 0.049) suggesting palmitate did not affect autophagosome formation, but instead impeded autophagosome clearance. Overall, a lower formation of autophagosomes partially contributed to lower LC3II/LC3I abundance with high‐dose insulin.

### Insulin‐mediated suppression of autophagosome formation required mTORC1

We investigated the requirement of mTORC1 for insulin‐mediated suppression of autophagy in L6 myotubes treated with rapamycin, an inhibitor of mTORC1. Rapamycin treatment resulted in a 40% reduction in the ability of insulin to suppress LC3II/LC3I accumulation after removal of serum (Fig. 5D). During bafilomycin treatment, rapamycin treatment resulted in a 56% reduction in the ability of insulin to suppress LC3II/LC3I accumulation after removal of serum (Fig. 5D). These results demonstrate a partial requirement of mTORC1 for insulin‐mediated suppression of autophagy, and the contribution of mTORC1 appears to be regulating the formation of autophagosomes.

## Discussion

The purpose of this study was to determine if skeletal muscle autophagy remains responsive to the suppressive effects of insulin despite the development of insulin resistance to glucose metabolism. Our primary findings indicate that insulin‐resistant mice remained responsive to the inhibitory action of insulin on autophagy as evidenced by similar autophagosome abundance during basal and hyperinsulinemic conditions in the HFD and LFD groups even though the HFD group had lower insulin‐stimulated glucose disposal. Autophagosome abundance declined to a similar level in response to high insulin and high glucose clamp conditions in both LFD and HFD groups. The autophagy response occurred alongside higher insulin levels in the HFD group compared to LFD and suggests a reduced sensitivity to insulin action. Across all clamp conditions, mitochondrial‐associated LC3II was greater in the HFD compared to the LFD group indicating a greater localization of autophagosomes to mitochondria. The mTORC1 pathway remained activated during high insulin conditions and cell culture experiments, despite insulin resistance, indicating mTORC1 activation may function as an intact signal to suppress formation of autophagosomes, even during high lipid conditions. Overall, our results indicate that autophagy remained responsive to the suppressive effects of insulin in obese mice that had skeletal muscle insulin resistance to glucose metabolism.

Insulin resistance is characterized by a decrease in insulin action and is commonly measured as a decrease in skeletal muscle glucose uptake per given concentration of insulin (Rizza et al. 1981a). Yet the resistance to insulin may not be consistent across pathways. For example, whole‐body protein breakdown was decreased under basal or hyperinsulinemic clamp conditions in both insulin‐sensitive adults and patients with type 2 diabetes (Bell et al. 2006). The observation that one pathway (glucose uptake) can develop resistance to insulin, whereas another (protein breakdown) can remain responsive to insulin in skeletal muscle is analogous to insulin resistance in the liver by which insulin has decreased ability to suppress gluconeogenesis but retains stimulation of de novo lipogenesis (Shimomura et al. 2000).

Impaired glucose uptake with insulin resistance can be defined by two components: the measured maximal response and the sensitivity to insulin (Rizza et al. 1981b). Our current reference to maximal response refers to the extent of autophagy suppression measured during physiological hyperinsulinemia and we note that further suppression may be possible. The maximal response of autophagy could be similar between mice consuming LFD or HFD concurrent with differences in sensitivity. To test these two components of insulin resistance, we performed mouse clamps and cell culture experiments at lower and higher insulin doses. The LFD and HFD mice had similar changes in autophagosome abundance during euglycemic and hyperglycemic clamps, but the HFD group had higher insulin concentrations than the LFD group. On the basis of the difference in insulin between the two diet groups, we cannot exclude the possibility of decreased sensitivity of autophagy to insulin action despite similar maximal measured suppression.

The suppression of autophagy by insulin may contribute to the decreased markers of autophagy that have been previously shown in insulin‐resistant adults (Møller et al. 2017) and animal models (Liu et al. 2009; Yang et al. 2010). The lower markers of autophagy may not be due to an intrinsic defect in autophagy with disease but instead be an intact response to hyperinsulinemia and hyperglycemia. For example, skeletal muscle autophagy markers were suppressed in healthy controls but not adults with type 2 diabetes after a hyperinsulinemic‐euglycemic clamp that maintained blood glucose at a concentration below fasting (Kruse et al. 2015). Interestingly, the combination of hyperinsulinemia and hyperglycemia, when blood glucose was clamped to elevated fasting concentrations, suppressed autophagy markers in the adults with type 2 diabetes (Kruse et al. 2015). Instead of a defect in autophagy with type 2 diabetes, a chronic suppression of autophagy may occur in response to compensatory hyperinsulinemia, possibly in combination with hyperglycemia.

The HFD mice had greater insulin concentrations during euglycemic and hyperglycemic clamps and it is possible for a decrease in sensitivity of autophagy suppression to occur despite a similar maximal measured suppression. We considered a possible dose effect of insulin and, interestingly, higher concentrations of insulin were required in both mouse and cell culture models to decrease autophagosome abundance. These findings suggest a threshold concentration may be required for insulin‐mediated suppression of autophagy, even though skeletal muscle glucose uptake was lower at both insulin concentrations in the HFD group. Previous work has demonstrated insulin resistance to protein turnover concurrently with insulin resistance to glucose uptake. In adults with type 2 diabetes, basal rates of skeletal muscle proteolysis were suppressed compared to healthy controls, but rates of proteolysis were not further suppressed with insulin stimulation (Denne et al. 1995). This is consistent with the concept of anabolic resistance of skeletal muscle protein synthesis to exercise in obesity (Nilsson et al. 2013).

Autophagy is an important component of mitochondrial protein turnover and is required to maintain skeletal muscle mass and mitochondrial abundance (Masiero et al. 2009). Autophagy is a dynamic process and the use of autophagy inhibitors is necessary to quantify flux (Mizushima and Yoshimori 2007). For in vivo measures, we were limited to using static markers of autophagy to avoid potential influence of inhibitors on glucose kinetics during the insulin clamps. We used mitochondrial localization of autophagy markers as a more targeted assessment of autophagy. Compared to low‐fat feeding, high‐fat feeding increased mitochondrial localized LC3II across all clamp conditions and suggests a preferential targeting of the autophagy machinery to the mitochondria. These results could illustrate a compensatory elevation in autophagosome abundance for removal of damaged proteins, as high‐fat feeding is known to result in the accumulation of damaged mitochondria (Laker et al. 2014), again suggesting there is not an intrinsic defect in the autophagy machinery. A methodological consideration is that the presence of lysosomes in the mitochondrial‐enriched fraction is an indicator of mitophagy (O'Leary et al. 2012), but is susceptible to contamination by fractionation procedures and may increase as a result of overall increased autophagosome abundance. Since basal autophagosome abundance was similar between the LFD and HFD groups, the greater association in the mitochondrial fraction in HFD is consistent with increased mitophagy.

Activation of mitophagy, particularly through Ulk1 signaling, appears to be required for the reformation of the mitochondrial network and functional recovery after skeletal muscle injury (Call et al. 2017). Such an observation demonstrates the essential role of autophagy in the remodeling of the mitochondrial proteome. We previously reported an increase in mitochondrial protein synthesis rates along with increases in LC3II abundance (Newsom et al. 2017), indicating HFD may increase autophagy machinery and mitochondrial protein turnover. The abundance of mitochondrial‐located LC3II also decreased with high insulin conditions, indicating a suppression of mitophagy in both insulin‐sensitive and insulin‐resistant mice. A chronic suppression of mitophagy as a result of hyperinsulinemia may account for increases in mitochondrial protein abundance with high‐fat feeding (Hancock et al. 2008; Newsom et al. 2017).

Signaling through mTORC1 could be an intact pathway to suppress autophagy when insulin signaling is dampened with obesity (Khamzina et al. 2005). mTORC1 exerts global inhibition on autophagy by phosphorylating an inhibitory site (Ser^757^) on Ulk1 (Ganley et al. 2009), which was increased in the HI/HG clamp along with phosphorylation of mTORC1 at the activating site (Ser^2448^). Inhibition of mTORC1 dampened the insulin effect in cultured myotubes, demonstrating a partial dependence on mTORC1 for insulin suppression of autophagy. These data are consistent with observations that rapamycin treatment blunts the refeeding‐induced suppression of LC3II abundance in mouse skeletal muscle (Naito et al. 2013).

We considered if the insulin‐mediated suppression of autophagy resulted from altered formation or clearance of autophagosomes. In the presence of an inhibitor of clearance, a change in autophagosome abundance can represent changes in formation. The suppressed autophagosome abundance with high‐dose insulin persisted in the presence of an inhibitor of autophagosome clearance, suggesting that the suppression of autophagy by insulin is at least partially mediated through reduced formation of autophagosomes. Palmitate treatment tended to increase autophagosome abundance under the different insulin doses but this effect was diminished when autophagosome degradation was inhibited. These results support that over‐abundance of lipids did not interfere with the insulin‐mediated suppression of autophagosome formation and instead suggest fatty acids may impede autophagosome clearance. This is consistent with findings that palmitate treatment impairs lysosomal enzyme activity and autophagosome degradation in cardiomyocytes (Jaishy et al. 2015).

There are additional considerations for the design and results of the current study. The HFD group had higher rates of energy expenditure and yet their physical activity was lower compared to the LFD group. Mice were clamped during the light cycle, their normal resting/fasting conditions, and there is likely a low contribution of physical activity to the current autophagy measures. While exercise is a known stimulus of autophagy (Laker et al. 2017), physical activity could not account for major differences in autophagy in the current study. The autophagy measurements are restricted to a single time point in the mice after 2 h of insulin and glucose stimulation. Therefore, our cell culture model was designed to recapitulate the conditions of the two‐hour clamp. As such, there could be transient changes to insulin and autophagy signaling that are not captured in our experimental time frame.

In conclusion, insulin‐resistant mice remained maximally responsive but may be less sensitive to the inhibitory action of insulin on autophagy activation, and mTORC1 is potentially an intact pathway for suppressing autophagy. The physiological consequences of autophagy suppression may be changes to protein abundance, particularly of mitochondria, during insulin resistance or type 2 diabetes as a result of chronic hyperinsulinemia. A lower degradation of mitochondrial proteins may contribute to the increased mitochondrial proteins that have been reported with obesity (Turner et al. 2007). Furthermore, autophagy is a necessary degradation pathway to avoid the accumulation of damaged proteins, such as advanced‐glycation end‐products that can occur with obesity (Mastrocola et al. 2015). Insulin‐mediated suppression of autophagy may contribute to accumulation of damaged mitochondrial proteins with poor function. Future considerations include determining if mitochondrial proteins are specifically targeted for degradation by autophagy and account for alterations in mitochondrial metabolism during obesity.

## Conflict of Interest

None declared.
